# Osteogenesis of Adipose-Derived and Bone Marrow Stem Cells with Polycaprolactone/Tricalcium Phosphate and Three-Dimensional Printing Technology in a Dog Model of Maxillary Bone Defects

**DOI:** 10.3390/polym9090450

**Published:** 2017-09-15

**Authors:** Jeong Woo Lee, Seung Gyun Chu, Hak Tae Kim, Kang Young Choi, Eun Jung Oh, Jin-Hyung Shim, Won-Soo Yun, Jung Bo Huh, Sung Hwan Moon, Seong Soo Kang, Ho Yun Chung

**Affiliations:** 1 Department of Plastic and Reconstructive Surgery, School of Medicine, Kyungpook National University, Daegu 41944, Korea; jeongwoo@hanmail.net (J.W.L.); choo870818@naver.com (S.G.C.); patraqushe@naver.com (H.T.K.); prschoi@gmail.com (K.Y.C.); fullrest74@hanmail.net (E.J.O.); 2 Cell & Matrix Research Institute, School of Medicine, Kyungpook National University, Daegu 41944, Korea; 3 Department of Mechanical Engineering, Korea Polytechnic University, Siheung-Si 15073, Korea; happyshim@kpu.ac.kr (J.-H.S.); wsyun@kpu.ac.kr (W.-S.Y.); 4 Department of Prosthodontics, Dental Research Institute, Institute of Translational Dental Science, School of Dentistry, Pusan National University, Yangsan-Si 50612, Korea; neoplasia96@hanmail.net; 5 Department of Medicine, School of Medicine, Konkuk University, Seoul 05029, Korea; sunghwanmoon@kku.ac.kr; 6 College of Veterinary Medicine, Chonnam National University, Gwangju 61186, Korea; vetkang@chonnam.ac.kr

**Keywords:** adipose-derived stem cells, bone marrow stem cells, osteogenesis, 3D-printed scaffold, polycaprolactone, tricalcium phosphate, dog, maxillary bone

## Abstract

Bone graft material should possess sufficient porosity and permeability to allow integration with native tissue and vascular invasion, and must satisfy oxygen and nutrient transport demands. In this study, we have examined the use of three-dimensional (3D)-printed polycaprolactone/tricalcium phosphate (PCL/TCP) composite material in bone grafting, to estimate the scope of its potential application in bone surgery. Adipose-derived stem cells (ADSCs) and bone marrow stem cells (BMSCs) are known to enhance osteointegration. We hypothesized that a patient-specific 3D-printed solid scaffold could help preserve seeded ADSCs and BMSCs and enhance osteointegration. Diffuse osteogenic tissue formation was observed by micro-computed tomography with both stem cell types, and the ADSC group displayed similar osteogenesis compared to the BMSC group. In histological assessment, the scaffold pores showed abundant ossification in both groups. Reverse transcription polymerase chain reaction (RT-PCR) showed that the BMSC group had higher expression of genes associated with ossification, and this was confirmed by Western blot analysis. The ADSC- and BMSC-seeded 3D-printed PCL/TCP scaffolds displayed promising enhancement of osteogenesis in a dog model of maxillary bone defects.

## 1. Introduction

Charles W. Hull, who founded 3D Systems in the United States in 1986, invented the world’s first three-dimensional (3D) printer. Since then, 3D printing has revolutionized a variety of manufacturing technologies, as well as tissue engineering and regenerative medicine [1]. Due to its innovative promise, 3D printing technology has attracted increasing interest in recent years, including its use in human organ and tissue development [2].

Tissue engineering is an important medical technology that combines biological and engineering techniques for the restoration of damaged or missing tissues or organs. Materials used to construct scaffolds for tissue engineering should have biocompatibility and biodegradability, and mechanical properties that can be supported in the body. In addition, an artificial scaffold prepared according to the characteristics of the transplantation site should allow differentiation and proliferation of cells attached to the pore [3,4,5,6]. The production of scaffolds using various materials has been an area of active study in tissue engineering.

Based on computed tomography (CT) or magnetic resonance imaging (MRI) data, it is possible to make patient-customized scaffolds using 3D printing technology. An advantage of this approach is that the mechanical properties, pore size, and porosity of the scaffold can be controlled. In addition, the excellent internal connective structure formed in the 3D shape improves the penetration and differentiation of the cells into the scaffolding, resulting in excellent biocompatibility. This technology is widely used for the reconstruction of ears and craniofacial defects in plastic surgery [7,8,9,10,11].

We have previously published literature introducing blended polycaprolactone (PCL) and tricalciumphosphate (TCP) as a 3D printable material for guided bone regeneration [12]. The blended TCP played a role in improving PCL’s deficient bone regeneration ability.

Stem cells are cells that can infinitely regenerate over a lifetime, and can differentiate into various cell types in response to specific biological signals and external stimuli. Techniques for differentiating mesenchymal stem cells (MSCs) isolated from bone marrow (bone marrow stem cells, BMSCs) and adipose tissue (adipose-derived stem cells, ADSCs), into chondrocytes, osteoblasts, and muscle cells are well established, and these cells can be used in tissue engineering to regenerate various tissues such as cartilage, bone, and muscle [13,14,15,16,17].

MSCs are the main constituents of bone marrow stromal cells, and are the precursors of bone, cartilage, muscle, and connective tissue [18]. Immunologically, they are rarely rejected, and proliferate readily without significant apoptosis [19]. Because of this nature, bone marrow-derived stem cells are of interest in biotechnology. ADSCs have also been actively studied in many fields because of their ease of acquisition, storage and handling of cells; however, ethical issues remain to be overcome [20,21,22].

We hypothesized that ossification between normal bone and a 3D-printed scaffold is an important factor in the reconstruction of maxillary bone defects, and that substances secreted from ADSCs and BMSCs could enhance osteointegration and thus increase both the degree of contact between the normal bone and the 3D-printed scaffold and bone formation inside the scaffold. This study was conducted to investigate the use of polycaprolactone/tricalcium phosphate (PCL/TCP) scaffolds, which are 3D-printed with a mixture of PCL, a polymeric material, and TCP, a bioceramic material, with ADSCs and BMSCs in bone regeneration in a dog model of maxillary bone defects.

## 2. Methods

### 2.1. Experimental Materials

#### 2.1.1. Fabrication and Characterization of 3D-Printed PCL/TCP Scaffolds

Biodegradable PCL (Evonik Industries AG, Essen, Germany) and TCP (Sigma-Aldrich, St. Louis, MO, USA) were used as materials for the scaffolds. PCL was dried at 105 °C for 1 day before use. TCP had a particle size of 100 nm or less. The 3D defect model was created by commercial 3D medical image editing software (Mimics, Materialize NV, Leuven, Belgium), and customized to maxilla defect of animals used in this study. A steel syringe containing the PCL/TCP mixture was equipped to a 3D printer and maintained at 120 °C. The molten PCL/TCP mixture was precisely dispensed through stainless steel nozzle with diameter of 300 µm. The inner microstructure and surface composition were characterized by field-emission scanning electron microscope (FE-SEM, S-4700, Hitachi Co., Ltd., Tokyo, Japan) and energy dispersive spectrometer (EDS) analyses (INCA Energy 350, Oxford Instruments, Oxford, UK).

#### 2.1.2. Harvest of ADSCs from Beagles

Adipose tissues obtained from the abdominal cavity of the beagles were washed with an equal volume of phosphate buffered saline (PBS). Then, enzymatic degradation was carried out at 37 °C for 30 min using 0.075% collagenase type I (Worthington Biochemical, Lakewood, NJ, USA). To remove debris from the connective tissues, the degraded tissue was filtered and the supernatant of the adipocyte layer was separated. The cell suspension was centrifuged for 10 min at a force of 200× *g*. Contaminating erythrocytes were removed by the addition of erythrocyte lysis buffer (Sigma-Aldrich, St. Louis, MO, USA) at pH 7.3. The stromal cells were rinsed twice with PBS, and the ADSCs were collected.

#### 2.1.3. Harvest of BMSCs from Beagles

Under general anesthesia, the humerus of each beagle was punctured with an 18-gauge injection needle and 1 mL of bone marrow tissue was collected with a 1 mL syringe. The collected bone marrow aspirates were combined with Histopaque (Sigma-Aldrich, St. Louis, MO, USA) at a ratio of 1:1 in a 15-mL tube and centrifuged at 2000 rpm for 30 min. After centrifugation, the white layer was collected in a 15-mL tube, diluted 10-fold in PBS, and then centrifuged at 2000 rpm for 10 min. After aspiration of the supernatant, the cells were again diluted 10-fold in PBS and centrifuged as above to collect BMSCs.

#### 2.1.4. Experimental Animals and Treatment Groups

Six healthy beagles, each 12 months old and weighing 9 kg, were included in the study. Prior to the 3D printing model experiment, spiramycin and metronidazole were injected under general anesthesia, the teeth were scaled, and the left molar and premolar were removed. Prior to implanting 3D-printed PCL/TCP scaffolds, the dogs were divided into two groups (*n* = 3 in each group) for application of ADSCs and BMSCs.

ADSCs and BMSCs were prepared at 1 μg/mL and injected into the 3D-printed PCL/TCP scaffolds in 0.01-mL increments in 10 areas of uniform distribution. Then, the 3D-printed PCL/TCP scaffolds were fixed in the maxillary bone defects of the beagles.

### 2.2. Experimental Methods

Thiopental sodium (Pentothal; Choong Wae Pharmacy, Seoul, Korea) was injected intravenously and general anesthesia was performed with halothane (Ilsung-halothane; Ilsung Pharmaceuticals, Seoul, Korea). During the operation, Lactated Ringer’s Solution and 1 g of cephalosporin were administered. After exposure to anesthesia, the fur on the left side of the beagle’s face was removed and the skin was disinfected with Betadine and alcohol. The maxillary bone was exposed through a skin incision from the zygomatic arch to the infraorbital foramen, and the maxillary bone defect was created from the infraorbital rim to the zygomatic arch in accordance with the 3D-printed scaffold prepared before surgery. The infraorbital nerve was preserved.

**Figure 1:**
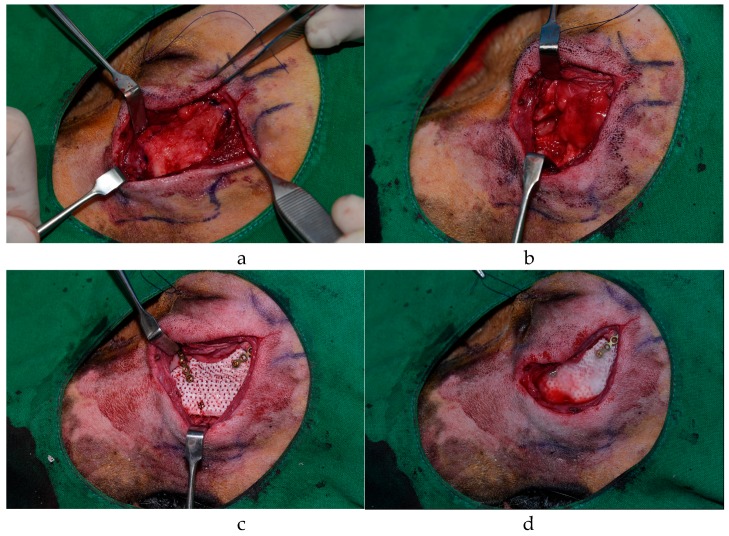
Experimental design: (**a**) exposed maxillary bone in a beagle model; (**b**) the defect was made; (**c**) the polycaprolactone/tricalcium phosphate (PCL/TCP) scaffold was fixed by plate and screw; and (**d**) fibrin glue seeding after adipose-derived stem cells (ADSCs) or bone marrow stem cells (BMSCs) were applied.

First, the bottom pores of the 3D-printed PCL/TCP scaffolds were blocked with fibrin glue. ADSCs and BMSCs were injected into the pores according to the experimental group, and then fibrin glue was applied to the exposed outer pores to prevent cell leakage. The prepared 3D-printed scaffold was inserted into the defect, fixed with a plate and screw, and the skin was sutured (Figure 1). Both groups were dressed once a day after surgery, and stitches were removed 7 days after surgery. All experiments conducted in this study were approved by the Institutional Animal Care and Use Committee of Chonnam National University (Approval No. CNU IACUC-YB-2016-43, The approval date is 28 September 2016) and followed their recommendations.

### 2.3. Experimental Evaluation

#### 2.3.1. Evaluation using 3D CT

To evaluate the ossification of the implanted 3D-printed scaffold, CT images of coronal, axial, and sagittal views were taken immediately after surgery, and 4, 8, and 12 weeks postoperatively. The ossification activity was determined by calcification of the marginal area, increased bone density, and decreased internal pore size. The level of tissue density depending on the time was analyzed with the Hounsfield unit (HU), which is a quantity commonly used in CT imaging. The HU value of the tissue in the pore was randomly measured at 5 sites for each individual, and the average values were obtained (Mimics Innovation Suite 17.0 64-bit version; Materialise NV, Leuven, Belgium). The measured HU values were normalized and calculated. To measure the extent of ossification, 3D-CT reconstruction was used.

#### 2.3.2. Histological Evaluation

For histological evaluation, all beagles were euthanized 12 weeks after 3D-printed scaffold implantation and the grafts were harvested, including 1 cm of marginal bone tissue. Tissues were taken from three normal bone/scaffold junctions and the center of one scaffold, and each was divided into three pieces. One third was fixed in 10% neutral formalin, and paraffin embedded tissue sections were stained with Hematoxylin and Eosin (H&E) and Goldner’s trichrome (GT). New ossification of the entire graft, the thickness and extent of periosteum produced, the degree of bone ingrowth into the pores, the infiltration of inflammatory cells into the graft, and the formation of collagen fibers were observed with an optical microscope (SkyScan 1173; Bruker microCT, Kontich, Belgium). Each slide was measured for optical density using an image analysis program (iSolution Lite; Image & Microscope Technology Inc., Vancouver, BC, Canada). In each group, the optical density of normal bone, tissue in the pore, and soft tissue around the scaffold including the periosteum were measured randomly at 5 sites for each tissue sample, and average values were obtained.

#### 2.3.3. Reverse Transcription Polymerase Chain Reaction (RT-PCR)

One third of each collected tissue was frozen in liquid nitrogen, and after grinding, total RNA was isolated using TRIzol (Thermo Fisher Scientific, Waltham, MA, USA). RNA was reverse transcribed to cDNA using a RevertAid First Strand cDNA Synthesis Kit (Thermo Fisher Scientific, MA, USA) and polymerase chain reaction (PCR) was performed using an AccuPower RT-PCR kit (Bioneer, Daejeon, Korea). Type 1 collagen (COL1), osteocalcin (OCN), runt-related transcription factor 2 (RUNX2) and glyceraldehyde-3-phosphate dehydrogenase (GAPDH) expression were measured by RT-PCR (Table 1).

#### 2.3.4. Western Blot Analysis

Proteins were extracted from obtained skin tissue by PRO-PREP kit (iNtRON Biotechnology, Seoul, Korea) and protein concentration was determined with BCA method. Proteins and 5X Sodium dodecyl sulfate polyacrylamide gel electrophoresis (SDS-PAGE) loading buffer were mixed and boiled at 95 °C for 5 min before incubation on ice. This protein was separated with 10% SDS-polyaclamide gel at 80~100 V for 2 h and transferred to nitrocellulose membrane at 100 V for 70 min on ice. Membranes were blocked with 5% skim milk at room temperature for 1 h and probed with primary antibody such as OCN (ab13420, 1 µg/mL; Abcam, Cambridge, MA, USA), COL1 (ab6308, 1 µg/mL; Abcam, Cambridge, MA, USA), RUNX2 (ab23981, 1 µg/mL; Abcam, Cambridge, MA, USA), β-actin (ab8226, 1:10000; Abcam, Cambridge, MA, USA). β-actin was used as a housekeeping protein. After incubating the membranes with antibody solution overnight at 4 °C, the membranes were washed with Tris buffered saline with Tween-20 (pH 7.4) and probed with secondary antibody for 1 hour at room temperature. Membrane was then exposed to ECL. The blots were followed immediately by exposure to X-ray film and the bands were quantified using ImageJ software (version 1.45s; U.S. National Institute of Health, Bethesda, MD, USA).

### 2.4. Statistical Analysis

Statistical analysis was performed with SPSS Ver. 22.0. (IBM Corp., Armonk, NY, USA). A subgroup analysis was performed using the paired *t*-test. Statistical significance was set at *p* < 0.05.

## 3. Results

### 3.1. Characterization of the 3D-Printed Scaffolds

**Figure 2:**
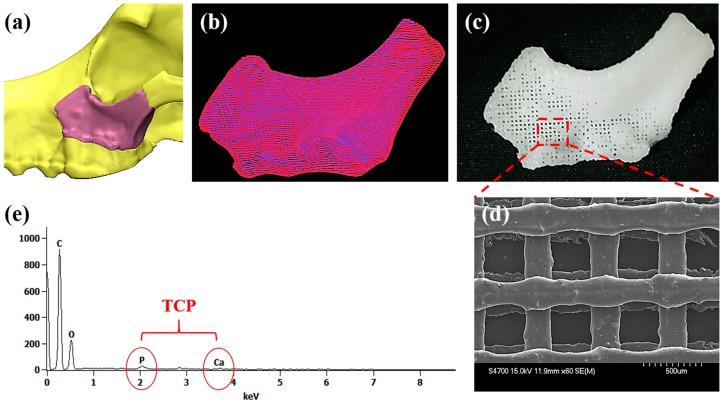
3D printing procedure and characterization: (**a**) 3D reconstruction of the defect; (**b**) printing path generation; (**c**) the photograph of the printed scaffold; (**d**) SEM image; and (**e**) EDS results.

For fabrication of the customized scaffold, 3D defect was reconstructed using Mimics software (Figure 2a). The 3D defect model was converted into printing path data by automated printing path generation algorithm (Figure 2b). Using the printing path data, the customized scaffolds were fabricated via 3D printer (Figure 2c). The line width and pore size of the printed scaffolds were 300 and 400 µm, respectively (Figure 2d). The EDS analysis was used to confirm exposure TCP onto the printed strut. As shown in Figure 2e, there were calcium (Ca) and phosphate (P) elements detected on PCL/TCP scaffold surface, indicating TCP powder was fully incorporated within the scaffold.

### 3.2. Evaluation of Results Using 3D CT

#### 3.2.1. Results from Coronal, Axial, and Sagittal CT Views

In both groups, ossification was not observed until four weeks after surgery. After eight weeks, the bone density along the scaffold margins was similar to that of normal bone. This can be regarded as progression of ossification along the margin, and showed a marked increase in density after 12 weeks. This suggests that ossification of the margin continuously increased for up to 12 weeks after surgery.

**Figure 3:**
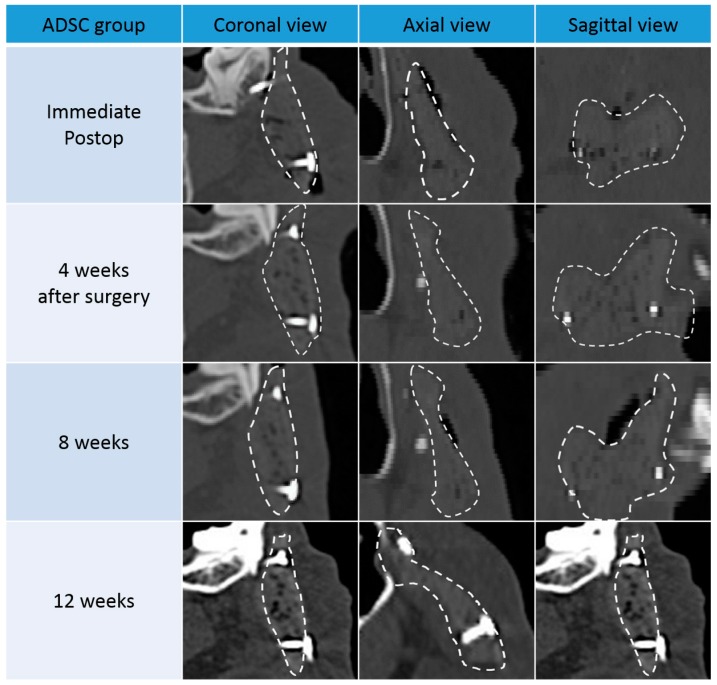
Computed tomography (CT) images of coronal, axial, and sagittal views from the adipose-derived stem cell (ADSC) group. Images were acquired directly after surgery and 4, 8, and 12 weeks later.

**Figure 4:**
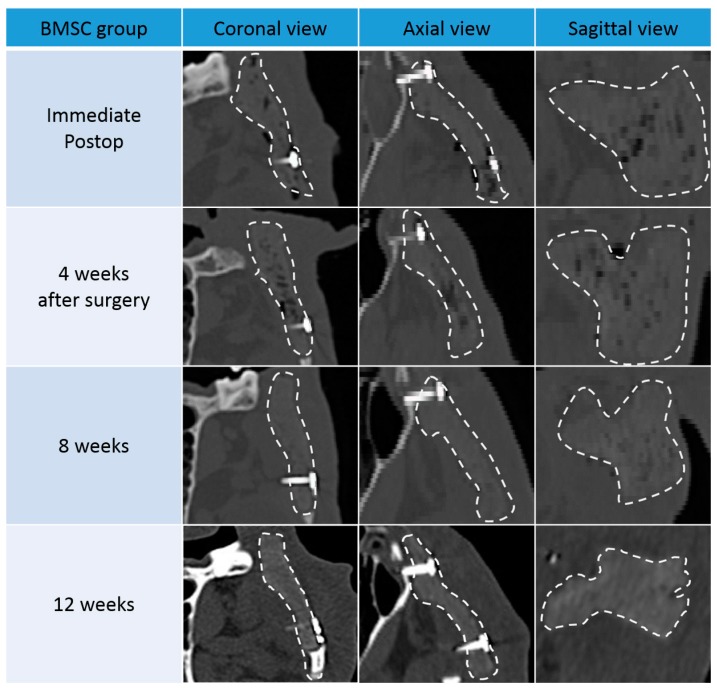
Computed tomography (CT) images of coronal, axial, and sagittal views from the bone marrow stem cell (BMSC) group. Images were acquired directly after surgery and 4, 8, and 12 weeks later.

In both groups, the size and number of pores inside the scaffold also decreased after eight weeks. However, the bone density of the scaffold was only partially increased, and did not reach the density of the surrounding normal bone. This suggests that most of the pores are formed by the increase in connective tissue and the inflammation reaction with the surrounding tissues and the ossification is partially occurring. After 12 weeks, pore size decreased further, and bone density was increased compared to eight weeks. However, at both 8 and 12 weeks, the BMSC group showed more pronounced marginal ossification and greater pore size reduction than the ADSC group (Figure 3 and Figure 4).

**Figure 5:**
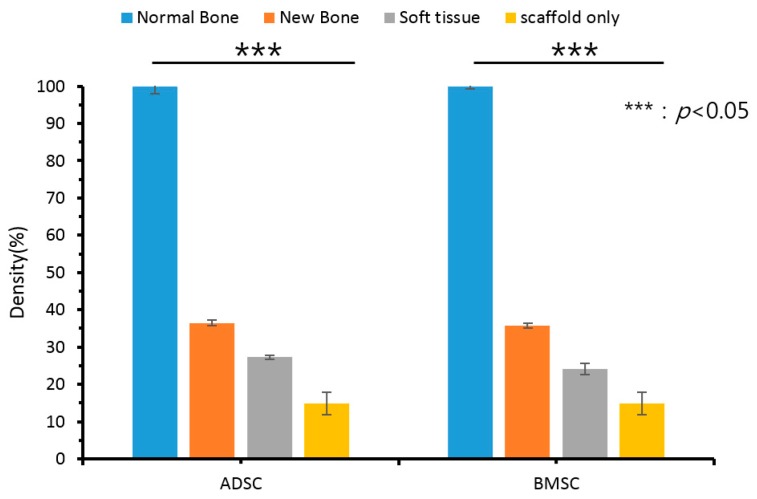
Gray values of normal bone, new bone, and soft tissue from the adipose-derived stem cell (ADSC) and bone marrow stem cell (BMSC) groups.

When the density of the normal bone was set to 100, the normalized density value of the new tissue (including the new bone) in the pores was 36.5 and 35.7 in the ADSC and BMSC groups, respectively, which was higher than the relative density of the periosteum around the scaffold (27.3 and 24.1 for the ADSC and BMSC groups, respectively; Figure 5). This suggests that ossification progressed sufficiently in the transplanted scaffolds in both groups. The ADSC group displayed a slightly higher density than the BMSC group. However, there was no significant difference between ADSC and BMSC groups. The normalized density of scaffold only (in vitro) was 15.

#### 3.2.2. Results from 3D CT

**Figure 6:**
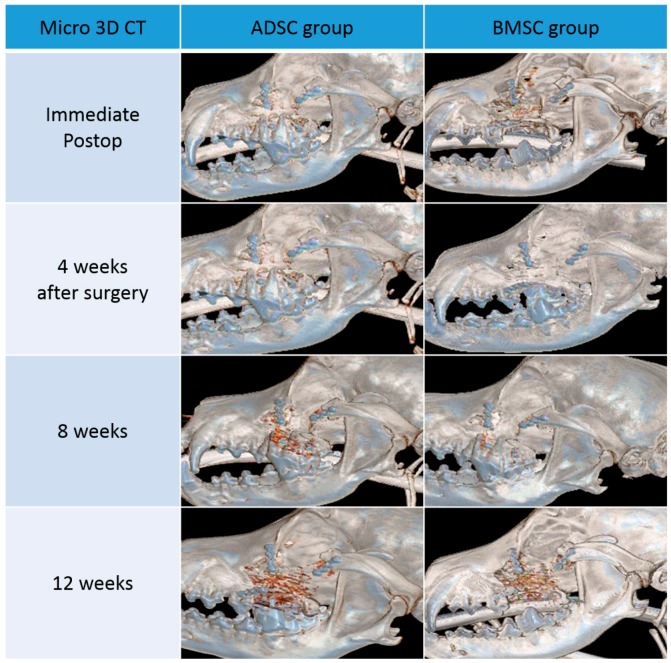
Three-dimensional (3D) computed tomography (CT) images of the adipose-derived stem cell (ADSC) and bone marrow stem cell (BMSC) groups. Images were acquired directly after surgery and 4, 8, and 12 weeks later.

In both groups, 3D CT did not display characteristic features of ossification until four weeks after surgery. After eight weeks, diffuse ossification could be observed inside the pores of the scaffold. A more prevalent ossification process was observed at 12 weeks in both groups. At 8 and 12 weeks, more pronounced ossification was observed in the ADSC group than in the BMSC group (Figure 6).

### 3.3. Histological Findings

**Figure 7:**
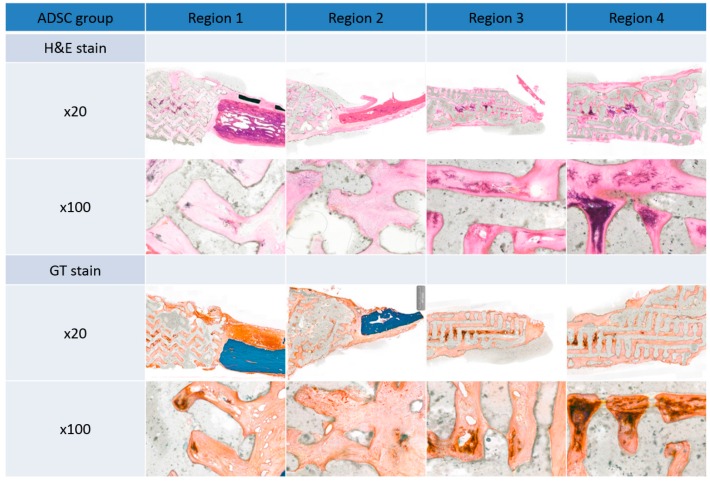
Histopathological analysis of the adipose-derived stem cell (ADSC) group 12 weeks after surgery by Hematoxylin and Eosin (H&E) and Goldner’s trichrome (GT) staining. Original magnification: ×20 and ×100.

**Figure 8:**
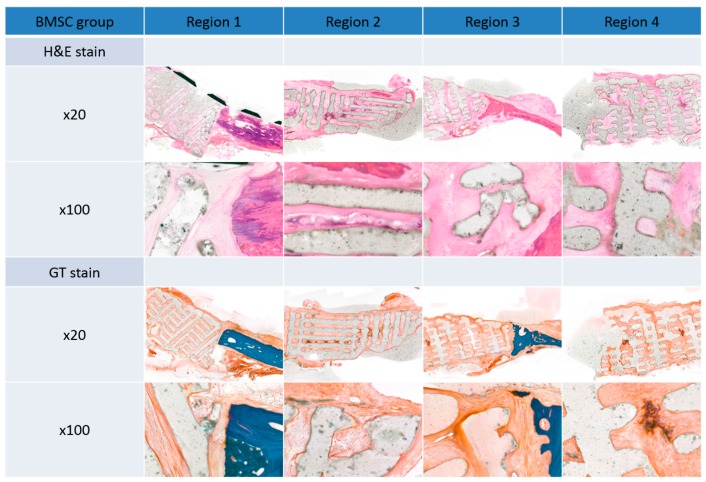
Histopathological analysis of the bone marrow stem cell (BMSC) group 12 weeks after surgery by Hematoxylin and Eosin (H&E) and Goldner’s trichrome (GT) staining. Original magnification: ×20 and ×100.

There was no gross rejection of the scaffold in either group. Upon histopathologic examination, connective tissue, such as the periosteum, was enclosed in the margin of the scaffold, and dense connective tissue was observed in the pore. These results demonstrate the excellent biocompatibility of the scaffolds. Some inflammatory findings were observed in the pore, which are thought to be due to inflammation before scaffold engraftment. Ossification was more pronounced in the center of the scaffold than in the margins. Ossification in the ADSC group was increased compared to the BMSC group (Figure 7 and Figure 8).

**Figure 9:**
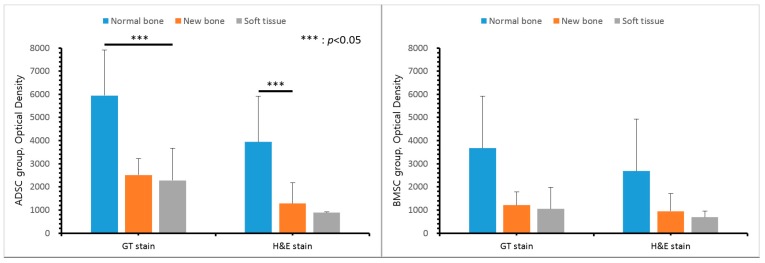
Optical density of Hematoxylin and Eosin stain (H&E) and Goldner’s trichrome (GT) staining.

The mean optical density values of normal bone, pore tissue, and soft tissues (including the periosteum) around the scaffold were obtained for the two groups. In GT and H&E staining, the optical density inside the pores was higher than that of the periosteum and other soft tissues around the scaffold, and was higher with GT than with H&E, however, there was no significant difference (Figure 9).

### 3.4. RT-PCR

**Figure 10:**
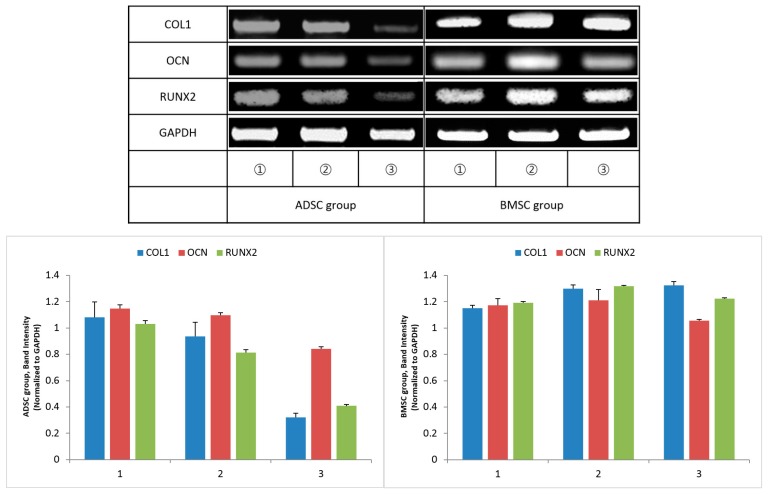
Expression of type 1 collagen (COL1), osteocalcin (OCN), runt-related transcription factor 2 (RUNX2) and glyceraldehyde-3-phosphate dehydrogenase (GAPDH) 12 weeks after surgery, measured by reverse transcription polymerase chain reaction (RT-PCR). ADSC, adipose-derived stem cell; BMSC, bone marrow stem cell.

Histological evaluation 12 weeks after surgery displayed ossification in the pore of the scaffold and an increase of fibrinogen in the periphery. The increase of fibrinogen was confirmed by an increase of COL1 gene expression, and ossification was confirmed by increases in OCN and RUNX2 gene expression. By RT-PCR, the band intensity of COL1, OCN, and RUNX2 was normalized to that of GAPDH. The degree of ossification-related gene expression was slightly increased in the BMSC group compared to the ADSC group, however, there was no significant difference (Figure 10).

### 3.5. Western Blot Analysis

**Figure 11:**
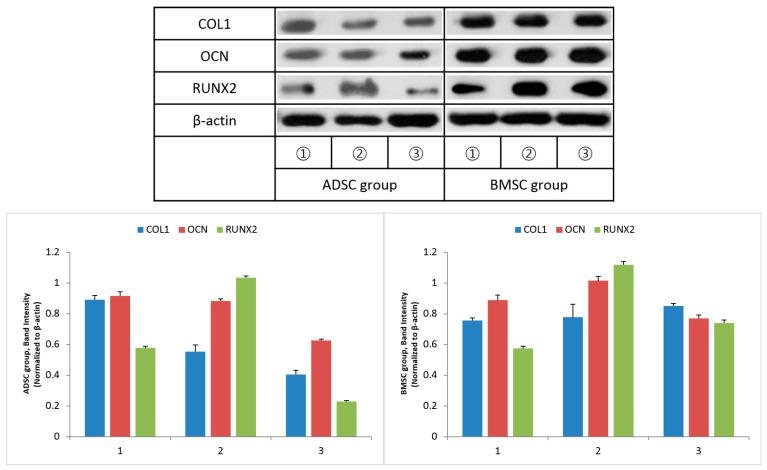
Expression of type 1 collagen (COL1), osteocalcin (OCN), runt-related transcription factor 2 (RUNX2) and β-actin 12 weeks after surgery, measured by Western blot analysis. ADSC, adipose-derived stem cell; BMSC, bone marrow stem cell.

Twelve weeks after surgery, expression of the ossification-related proteins COL1, OCN, and RUNX2 was increased in both groups. The expression of these proteins was higher in the BMSC group than in the ADSC group (Figure 11). In particular, the expression of COL1 was significantly higher in the BMSC than in the ADSC. The band intensity of COL1, OCN, and RUNX2 was normalized to that of β-actin.

## 4. Discussion

Due to its ease of use and adaptability, 3D printing technology is being rapidly developed and applied in various medical fields. For example, 3D printing technology can be used to visualize the complex deformities of congenital cardiovascular anomalies, to map intricate blood vessels during renal cancer surgery, and allow the development of approaches to intracranial tumor neurosurgery. These applications help surgeons analyze and evaluate the individual condition of the patient before surgery, aiding in the development of a surgical plan [23,24]. However, despite being an innovative technology that can be continuously developed in the medical field, there remains a lack of scientific data on its application.

With tissue engineering, a new era in bioengineering has begun. In general, tissue engineering involves three components: cells, scaffolds, and growth factors. Recently, 3D technologies have been used to develop cell and tissue printing technologies, allowing tissue engineering to go beyond conventional cell culture technology. Scaffold development and 3D cell culture both have remaining limitations, but it is expected that in the near future, 3D cell-printing technology will enable the simultaneous formation of 3D biological structures and scaffolds [25,26,27,28,29,30].

Currently, 3D printing technology is most often used in craniomaxillofacial surgery, and several challenges must be overcome for its widespread application. First, more specialized computer software is required. Pre-operative 3D printing is not easy to plan because it takes too much time to perform a computer simulation of a differentiated process. This requires more automated functionality and simulation via a variety of software.

Second, there must be a sufficient relationship between the pre-operative simulation and the actual operating environment, because the pre-operative simulation serves as a guide for the operation. For example, to apply 3D-printed titanium implants in surgery, bone must be resected precisely according to the preoperative plan, as 3D-printed implants are generally quite hard, and cannot easily be cut or bent.

Third, accuracy during measurement is very important. Due to technological advances, it is possible to obtain very thin CT images, but the combination of these images to form 3D images introduces error, particularly for very thin bones such as the orbital bones.

Finally, there is a possibility of artifacts due to metal materials. The use of CT scans in 3D printing for prosthetic dentistry, which mainly uses metal models, may be less accurate due to these artificial shadows. Tooth occlusion requires very fine 3D rendering, and CT may not provide images of adequate resolution because of these artifacts [31,32].

Despite these problems, 3D printing technology is attracting attention as a new medical technology. Using 3D printing, the medical industry could visualize various characteristics of individual patients and apply them to personalized medical care.

Scaffolds used in tissue engineering are generally made of natural and synthetic polymeric materials. Polymers derived from natural materials, animals, and human bodies have superior biocompatibility and are nontoxic. These include gelatin, collagen, fibrin, elastin, and alginate. Synthetic polymeric materials are relatively inexpensive, have excellent mechanical properties, and are either hydrolyzed in vivo or degraded by enzymes, making them ideal polymers for scaffolds. PCL, polylactide-co-glycolide, and polylactic acid (PLA) are typical synthetic polymer materials. In particular, various scaffolds for bone tissue regeneration are made using PLA and TCP, a bioceramic material. Since TCP is made of powder, it confers decreased mechanical strength to the scaffold in comparison with a solid polymer material that can be produced using heat, and is more difficult to form into 3D scaffold with excellent internal pore structure. To overcome this problem, many studies have fabricated scaffolds by mixing ceramic and polymeric materials [33,34,35,36,37].

The 3D printing system enables precise control and rapid production of desired 3D shape structures at the micron scale. It also has the advantage of determining the line width and line height of laminated materials according to pneumatic pressure, temperature, and moving speed. Generally, when a scaffold is produced by 3D printing with a mix of polymer and a bioceramic material, the ceramic material content is limited. Because of the difficulty of nozzle discharge with increased amounts of bioceramic powder, controlling the mixing method of the bioceramic powder is a very important aspect of scaffold development. In this study, a 3D printing system was used to fabricate 3D scaffolds from PCL mixed with β-TCP, and evaluated their effects on bone formation.

MSCs grow adherently in the form of fibroblasts in vitro, form cell clusters from single cells, and differentiate into osteocytes, adipocytes, and chondrocytes. Friedenstein first reported the presence of MSCs in the bone marrow in 1970 [38]. Subsequently, cells with similar characteristics have been reported in various tissues such as adipose, brain, spleen, liver, kidney, lung, bone marrow, muscle, thymus, and pancreas [39].

In order to improve osteointegration in bone grafting and the regeneration of bone defects, ADSCs and BMSCs have recently been used in tissue engineering. However, when harvesting BMSCs, complications such as pain and infection are likely to occur, and there is a limit to the amount of BMSCs taken. In contrast, ADSCs are readily available in large quantities under local anesthesia [13,14,15,16]. ADSCs are simpler to collect than BMSCs, with less donor morbidity, and adipose tissue provides more stem cells than bone marrow. However, previous studies have shown that BMSCs induce bone formation after differentiation into osteoblasts in vitro and in vivo, and thus provide favorable conditions for regeneration of bone tissue [40,41,42,43].

The use of scaffolds for the application of ADSCs and BMSCs to bone defects has many advantages. The scaffold serves as a bridge, allowing adequate time for stem cell survival and subsequent bone formation. Generally, the scaffold should be strong enough to withstand external impact, and the pores should disassemble after the bone has adequately regenerated. In this study, an artificial scaffold was prepared using biodegradable PCL/β-TCP, which is currently in clinical use as an osteoinductive material. β-TCP is highly compatible with bone morphogenetic protein 2 (BMP-2), a bone growth-promoting factor, and thus increases osteoinduction [44,45,46,47]. In addition, β-TCP degrades with time. These two properties are ideal for scaffolds used in bone regeneration.

To evaluate the bone regeneration ability of PCL/β-TCP scaffolds injected with ADSCs and BMSCs, 3D CT and histological analysis were performed after implantation of the scaffolds into maxillary bone defects in beagles. Diffuse osteogenic tissue formation was observed by micro-computed tomography with both stem cell types, and the ADSC group displayed similar osteogenesis compared to the BMSC group. RT-PCR and Western blot analysis confirmed increased expression of proteins associated with osteogenesis in both groups, with higher expression of COL1 of the BMSC group observed in Western blot. As a result, new bone formation inside the scaffold was effective in the reconstruction of maxillary bone defects in a dog model using PCL/TCP biomaterials manufactured using a 3D printing system, with ADSCs and BMSCs. However, unlike other previous studies, gross and histological results suggested that ADSC treatment was superior to BMSC treatment.

These results suggest that ADSCs induce osteogenesis similar to BMSCs when 3D-printed PCL/TCP scaffolds are implanted in bone defects. Clinically, ADSCs have many advantages over BMSCs because ADSCs can be harvested easily, quickly, safely, and in relative abundance by lipoaspiration in the office-based setting. On the other hand, BMSCs harvest is an invasive procedure with low yield, which may impart donor site morbidity, such as pain, bleeding, hematoma, and deep infection. Therefore, if used with a 3D-printed PCL/TCP scaffold, ADSC can be a good alternative to BMSC to improve osteointegration in bone grafting and the regeneration of bone defects.

## Figures and Tables

**Table 1:** Sequences of primers used for reverse transcription polymerase chain reaction (RT-PCR).

Gene name	Sequence (5′-3′)
COL1-dog-F	CTCGTCACAGTTGGGGTTGA
COL1-dog-R	GGTGCAAGTATGAAGCGGGA
OCN-dog-F	AATTGCGCTCGAGCATCTCT
OCN-dog-R	ATTGCCACGGTTGCTACTGA
RUNX2-dog-F	GGCGGCTATAACTCTTCCCA
RUNX2-dog-R	ACGCAGCGGCTTTTTATTTCA

COL1, type 1 collagen; OCN, osteocalcin; RUNX2, runt-related transcription factor 2.
